# Evaluation of Inferior Turbinate Stroma with Ultrasound Elastography in Allergic Rhinitis Patients

**DOI:** 10.4274/balkanmedj.2016.1339

**Published:** 2017-08-04

**Authors:** Göksel Turhal, Sercan Göde, Ceyda Tunakan Dalgıç, Aytül Zerrin Sin, Erkan Kısmalı, İsa Kaya, Arın Öztürk, Özlem Göksel, Raşit Midilli, Kerem Öztürk, Bülent Karcı

**Affiliations:** 1 Department of Otolaryngology, Ege University School of Medicine, İzmir, Turkey; 2 Department of Allergy and Immunology, Ege University School of Medicine, İzmir, Turkey; 3 Department of Radiology, Ege University School of Medinice, İzmir, Turkey; 4 Department of Pulmonology, Ege University School of Medicine, İzmir, Turkey

**Keywords:** Sonoelastography, ultrasound elastography, allergic rhinitis, Fibrosis, inferior turbinate

## Abstract

**Background:**

Diagnosis of allergic rhinitis is primarily based on history, physical examination and allergy testing. A technique that noninvasively evaluates the soft tissue changes in the nasal mucosa of allergic rhinitis patients has not been defined.

**Aims:**

To assess nasal mucosal changes and measure the submucosal fibrosis in allergic rhinitis patients with sonoelastography.

**Study Design:**

Case control study.

**Methods:**

Eighty-eight turbinates of 44 patients were included in the study. There were 23 prick test positive allergic rhinitis patients. The control group constituted 21 patients. The rhinitis quality of life questionnaire and the visual analogue scale were applied to the allergic rhinitis patients. A higher visual analogue scale score indicated more severe allergic rhinitis symptoms. Sonoelastographic measurements were made from the lateral nasal wall. The propagation speed of sound waves was recorded in m/s. The presence of asthma and the type of allergic rhinitis (seasonal or perennial) was noted.

**Results:**

Ten patients had seasonal allergic rhinitis and thirteen patients had perennial allergic rhinitis. Six patients (26.1%) had accompanying asthma along with allergic rhinitis. The median visual analogue scale score was 7 (3-9) in allergic rhinitis patients. The median symptom duration was 7 (1-24) months. The median quality of life questionnaire score was 3.39 (1.68-5.43) points. The median sonoelastography scores of allergic rhinitis patients and healthy subjects were 2.38 m/s (0.9-4.47) and 2.42 m/s (1.62-3.50), respectively. Sonoelastographic measurements of seasonal and perennial allergic rhinitis patients did not differ significantly (p>0.05). The presence of asthma did not have a significant impact on the elastography measurements (p>0.05). However, regression analysis revealed a significant inverse correlation (coefficients: B=0.005, standard error=0.097, beta 0=0.008) between the visual analogue scale and sonoelastography scores (p<0.05).

**Conclusion:**

Sonoelastography was not suitable as a diagnostic tool in allergic rhinitis. Reduced sonoelastography scores were measured in more symptomatic patients. Higher visual analogue scale scores could be an indicator of disease severity.

Ultrasound elastography or sonoelastography is a relatively new technique for measuring tissue stiffness and assessing tissue fibrosis. This technique is currently being used for the evaluation of superficial tissues such as breast, thyroid gland, lymph nodes, muscle and visceral organs such as liver and kidney (1,2,3,4). The use of ultrasound elastography to evaluate inferior turbinates was demonstrated in a previous study and ultrasound elastography was found to be a reliable, repeatable, non-invasive and objective method (5). Ultrasound elastography involves no ionising radiation and thus is not harmful to human tissue. The first clinical application of this new technique was also reported in patients undergoing inferior turbinate reduction procedures (6), and tissue fibrosis caused by inferior turbinate cauterisation and radiofrequency ablation was measured (6). Allergic rhinitis (AR) is a clinical hypersensitivity disorder of the nasal mucosa and has a prevalence of 10-20% (7,8). It is well known that inflammatory mediators and proinflammatory cytokines cause various inflammatory reactions in the nasal mucosa. Seromucous gland proliferation and submucosal fibrosis has been shown in patients with a long history of AR (9). A technique that noninvasively evaluates the soft tissue changes in the nasal mucosa of the AR patients has not yet been developed.

The primary aim of this study was to assess nasal mucosal changes and measure the degree of submucosal fibrosis in AR patients using ultrasound elastography. The secondary aim of this study was to evaluate the diagnostic value of ultrasound elastography in AR patients, and to assess the correlation between elastography scores and symptom severity.

## MATERIALS AND METHODS

This study was conducted at the otolarynology, immunology and pulmonology departments of a tertiary academic center between January 2015 and October 2015. It was carried out in accordance with international ethical standards and the World Health Organisation Helsinki Declaration. The study was approved by the institutional review board (Approval identification #: 15-5/1). Informed consent was obtained from all of the subjects.

### Patient selection

Eighty-eight turbinates of 44 subjects were included in the study. There were 23 (46 turbinates) prick test-positive AR patients. Patients with a history of tobacco use, previous sinonasal surgery, recent sinonasal infection, chronic sinusitis, ciliary dysfunction, diabetes mellitus or cardiovascular disease were not included. The control group constituted 21 patients (42 turbinates) with no allergic, vasomotor or infectious rhinitis symptoms. The patients who were included in the study did not have clinical septal deviation that would interfere with sonoelastographic measurement. Twenty-five AR patients and 25 control subjects were initially planned, but two AR patients and four control subjects did not want to participate in the study and thus were excluded.

### Instrumentation

All of the US elastographic evaluation was performed using a Siemens, ACUSON S2000 (Siemens Medical Solutions USA, Inc. 685 East Middlefield Road Mountain View, CA 94043 USA) ultrasonography device. The 'Virtual Touch Tissue QuantificationTM' method based on the non-invasive 'acoustic radiation force imaging' method was used with the 9-4 MHz linear array probe.

### Procedure

**Figure 1:**
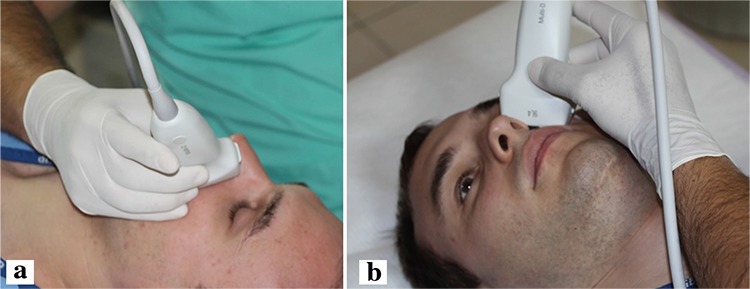
Patient and ultrasound probe positioning. The ultrasound elastography is performed from the lateral nasal wall.

AR was diagnosed with history and clinical examination including full otolaryngologic examination and nasal endoscopy. The diagnosis of AR was confirmed and supported with an intradermal skin prick test. The Rhinitis Quality of Life Questionnaire (RQLQ) and the visual analogue scale (VAS) were applied to the AR patients (10). A higher VAS score indicated more severe AR symptoms. Ultrasound elastographic measurements were made from the lateral nasal wall (Figure 1) according to a previously described technique (5). All of the measurements were made by the same radiologist in order to standardise the results. Ten valid measurements were performed each time for each turbinate. The propagation speed of sound waves was recorded in metres/second (m/s) and the median value of these ten measurements was used. Higher propagation speeds are associated with stiffer tissue, as the speed of sound waves is higher in a solid medium. The reverse is true for lower propagation speed values. The presence of asthma and the type of AR (seasonal or perennial) was noted.

### Outcomes

Sonoelastography measurements of the two groups were analysed and compared in order to assess the degree of fibrosis in AR patients compared to healthy subjects. The relationships of RQLQ and VAS scores with the sonoelastographic measurements were evaluated. Additionally the relationships of the duration of symptoms and the presence of asthma with the sonoelastography scores were assessed.

### Statistical analysis

Statistical analysis was performed using the software SPSS (IBM Corp. Released 2013. IBM SPSS Statistics for Windows, Version 22.0. Armonk, NY: IBM Corp.). Chi-square (X2) exact tests were used for the comparison of categorical data. Independent and paired sample t-tests were used for the analysis of parametric variables, while Wilcoxon and Mann-Whitney U tests were used for the analysis of non-parametric variables. The distribution patterns of the data were determined with the Shapiro-Wilk test. The distribution of the groups was non-parametric. Based on the distribution pattern of the data, correlation analysis was performed via Pearson or Spearman correlation analysis. Data were expressed as “mean ± standard deviation”, percent (%), minimum-maximum, odds ratio; 95% confidence interval and “median [interquartile range (IQR)]” where appropriate. A p value lower than 0.05 was considered as statistically significant.

## RESULTS

Forty-four patients with a mean age of 28.27 years (range 18-71, 21 female, 23 male) were included in the study. Ten patients had seasonal AR and 13 patients had perennial AR. Six patients (26.1%) had accompanying asthma along with AR.

The median VAS score was 7 (IQR=3, range 3-9) in AR patients. The median symptom duration was 7 months (IQR=7) (range 1-24 months). The median RQLQ scores were 3.39 points (IQR=1.93, range 1.68-5.43). The median sonoelastography scores of AR patients and healthy subjects were 2.38 m/s (IQR=0.89, range 0.93-4.47) and 2.42 m/s (IQR=1.02, range 1.62-3.50), respectively (Table 1). According to the independent samples t-test there was no statistically significant difference between AR patients and control subjects regarding sonoelastography scores (p>0.05).

Sonoelastographic measurements of seasonal and perennial AR patients did not differ significantly (p>0.05) (Table 2). Also the presence of asthma did not have a statistically significant impact on the elastography measurements (p>0.05). The severity of AR and the degree of AR symptoms were evaluated using the RQLQ and VAS scores. RQLQ scores were not correlated with sonoelastography scores (p>0.05). However regression analysis revealed a statistically significant correlation (coefficients: B=0.005, standard error=0.097, beta 0=0.008) between VAS scores and sonoelastography scores (p<0.049). The VAS score was inversely proportional to the sonoelastography score.

## DISCUSSION

Diagnosis of AR is based on history and physical examination. Diagnosis is also established and supported by in vivo and in vitro allergen tests. These tests also provide information about the offending allergen. Given the high costs and limited availability of allergic testing outside academic centres physicians in the primary healthcare setting rely on history and nasal examination (10). AR has a major impact on quality of life, sleep, school or work performance, and productivity. Besides history and nasal examination, questionnaires are used both in the diagnosis of AR and to measure the impact of AR on the patient.

AR is a type 1 hypersensitivity reaction in which re-exposure to specific antigens causes IgE-mediated histamine degranulation of basophils and mast cells. Histamine binds to histamine receptors and causes vasodilatation, thus increasing permeability (11). This increased permeability causes rhinorrhoea, sneezing and nasal congestion. Other released proinflammatory mediators, including IL-5, recruit eosinophils and leukotrienes and promote late phase inflammation. Most studies that investigate the tissue characteristics of nasal mucosa and submucosa focus on the presence of inflammatory cells. Lim et al. (12) reported significant increases of intraepithelial mononuclear cells and total cells in seasonally allergic subjects. Jacobson et al. (13) reported significant increases in submucosal eosinophils but not mast cells in pollen-sensitive seasonal rhinitis patients. All published studies assessing the histocytological characteristics of the nasal mucosa required a biopsy and thus were invasive.

In this study, tissue stiffness and fibrosis in inferior turbinates of AR patients were quantitatively compared with healthy subjects without the need for an invasive procedure. Fibrosis induced by electrocauterization and radiofrequency application were demonstrated and measured with sonoelastography in previous studies (5,6). The same sonoelastography technique was used in this study, however, a statistically significant difference in sonoelastography measurements of AR patients compared to the control group was not found (5,6). It is known that patients with a long history of AR develop submucosal tissue fibrosis, but this could not be detected in sonoelastographic measurements. This may be related to the median duration of symptoms in AR patients, which was seven months in this study - this duration was not sufficient to create the submucosal fibrosis that would have an effect on sonoelastography. It is difficult to find AR patients with a long duration of symptoms who have not undergone any form of therapy and this is a limitation of the present study. Controlled studies evaluating AR patients with longer symptom duration and assessing the effect of AR therapies on inferior turbinate stroma are warranted.

Seasonal and perennial AR patients have different characteristics related to their symptoms and duration of their symptoms, but the degree of tissue fibrosis measureable with the sonoelastography technique was not significantly different between these two group of patients.

AR and allergic asthma are related diseases that commonly co-exist. According to the united airway disease hypothesis, upper and lower airway diseases are both manifestations of a single inflammatory process (14). Asthma is present in 20 to 50% of patients with AR and rhinitis is present in up to 80% of patients with asthma (15,16,17). The relationship between the presence of asthma and the sonoelastography scores was evaluated in AR patients. However, the sonoelastography measurements of AR patients with asthma were not significantly different from those of AR patients without asthma.

The RQLQ is a questionnaire specifically planned to measure the impact of AR on patients and is validated for the Turkish language (18,19,20). The severity of the symptoms was evaluated using both the RQLQ and the VAS. We did not find a statistically significant correlation between RQLQ scores and sonoelastography measurements. However there was a statistically significant correlation between the VAS and the sonoelastography scores. This correlation was an inversely proportional correlation which means higher (worse) VAS scores were correlated with a less dense, probably more oedematous stroma (lower sonoelastography score) and this could well be a numerical explanation for increased congestion.

In conclusion, sonoelastography was not suitable as a diagnostic tool in AR. Reduced propagation speed of sonoelastography waves was recorded in more symptomatic patients. Higher VAS scores could be an indicator of disease severity. Sonoelastography might have diagnostic value in AR patients with acute exacerbation of nasal symptoms and in patients with active nasal congestion fullness. However, this needs to be elucidated in a new study with new patient groups.

## Figures and Tables

**Table 1:**
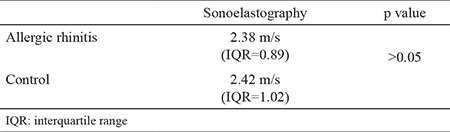
Sonoelastography measurements of allergic rhinitis patients and healthy subjects

**Table 2:**
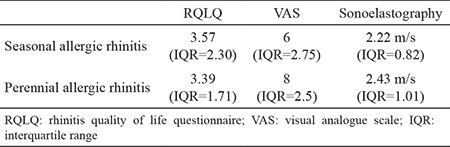
Median rhinitis quality of life questionnaire, visual analogue scale and sonoelastography scores of seasonal and perennial allergic rhinitis patients
